# 4-(Anthracen-9-yl)-2-phenyl-6-(pyridin-2-yl)pyridine

**DOI:** 10.1107/S1600536812012299

**Published:** 2012-03-28

**Authors:** Hao-Wei Wang, Jun Ren, Wen-Bo Ye, Jia-Xiang Yang

**Affiliations:** a Deparment of Chemistry, Anhui University, Hefei 230039, People’s Republic of China

## Abstract

In the title compound, C_30_H_20_N_2_, the anthracene ring system is approximately planar [maximum deviation = 0.035 (2) Å] and is nearly perpendicular to the central pyridine ring, making a dihedral angle of 75.73 (7)°. The terminal pyridine ring and the phenyl ring are oriented at dihedral angles of 8.11 (10) and 13.22 (10)°, respectively, to the central pyridine ring.

## Related literature

For applications of aromatic conjugated organic compounds, see: Nishihara *et al.* (1989 ▶); Mi *et al.* (2003 ▶); Roberto *et al.* (2000 ▶).
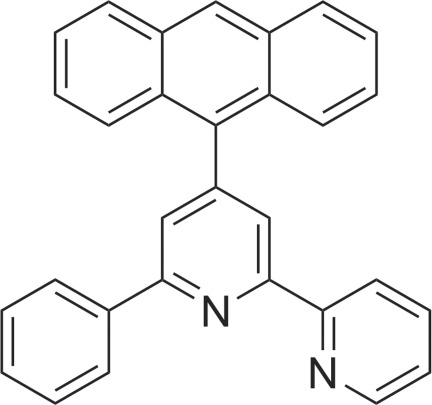


## Experimental

### 

#### Crystal data

C_30_H_20_N_2_
*M* *_r_* = 408.48
Monoclinic, 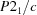

*a* = 12.6420 (3) Å
*b* = 14.8499 (4) Å
*c* = 11.8707 (3) Å
β = 104.006 (2)°
*V* = 2162.26 (9) Å^3^
*Z* = 4
Mo *K*α radiation
μ = 0.07 mm^−1^
*T* = 298 K
0.2 × 0.2 × 0.2 mm

#### Data collection

Bruker SMART 1000 CCD area-detector diffractometer
35644 measured reflections
4951 independent reflections
3128 reflections with *I* > 2σ(*I*)
*R*_int_ = 0.038

#### Refinement

*R*[*F* ^2^ > 2σ(*F* ^2^)] = 0.063
*wR*(*F* ^2^) = 0.215
*S* = 1.33
4951 reflections
289 parameters
H-atom parameters constrained
Δρ_max_ = 0.20 e Å^−3^
Δρ_min_ = −0.20 e Å^−3^

### 

Data collection: *SMART* (Bruker, 2007 ▶); cell refinement: *SAINT* (Bruker, 2007 ▶); data reduction: *SAINT*; program(s) used to solve structure: *SHELXTL* (Sheldrick, 2008 ▶); program(s) used to refine structure: *SHELXTL*; molecular graphics: *SHELXTL*; software used to prepare material for publication: *SHELXTL*.

## Supplementary Material

Crystal structure: contains datablock(s) I, global. DOI: 10.1107/S1600536812012299/xu5476sup1.cif

Structure factors: contains datablock(s) I. DOI: 10.1107/S1600536812012299/xu5476Isup2.hkl

Supplementary material file. DOI: 10.1107/S1600536812012299/xu5476Isup3.cml

Additional supplementary materials:  crystallographic information; 3D view; checkCIF report

## Figures and Tables

**Fig. 1.**
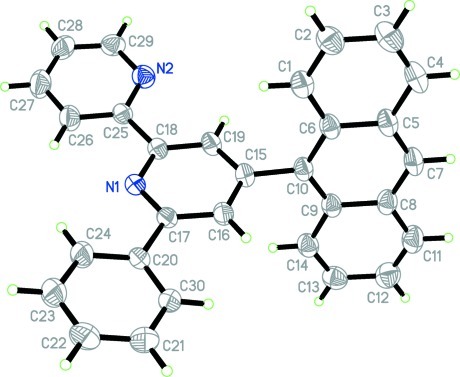
The molecular structure of the title molecule, with the atom-numbering scheme. Displacement ellipsoids are drawn at the 50% probability level.

**Fig. 2.**
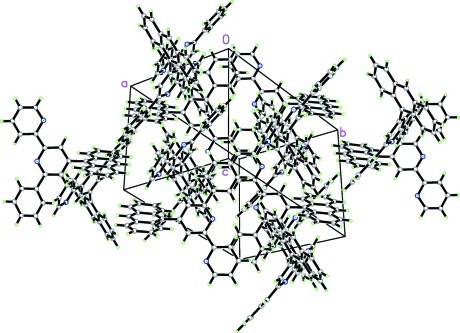
A packing diagram of (I).

## supplementary crystallographic information

### Comment

The aromatic conjugated organic compounds are investigated with great interest due to their potential applications in optical image processing, all-optical switching, organic light emitting diodes (OLEDs) and integrated optical devices (Nishihara *et al.*, 1989; Mi *et al.*, 2003; Roberto *et al.*, 2000). As a part of our continuing studies of the synthesis and characterization of optical materials, we have prepared a new anthracene derivative containing two pyridine rings and investigated its crystal structure.

The molecule structure of (I) is shown in Fig. 1. Two pyridine rings makes the dihedral angle of 8.11 (10)°. The anthracen moiety is almost planar, and make the dihedral angles of 75.73 (7)° and 67.84 (2)° with two pyridine rings, respectively.

### Experimental

3-(Anthracen-9-yl)-1-phenylprop-2-en-1-one (1.54 g, 5.0 mmol), 2-acetylpyridine (1.82 g, 15 mmol) and NaOH (0.20 g, 5.0 mmol) were crashed together with a pestle and mortar for 3 h. The light yellow powder was added to a stirred solution of ammonium acetate (15.4 g, 200.0 mmol) in ethanol (200 ml). The reaction mixture was heated at reflux. Thin layer chromatography analysis tracking reaction, evaporated solvent, extracted with dichloromethane, and dried to afford the product. It was purified by flash column chromatography on silica. Elution with petroleum/ethyl acetate (10:1) gave a white solid (yield; 1.3 g, 65%). Single crystals of (I) were grown by slow evaporation of a dichloromethane/ethyl acetate (1:1) solution.

### Refinement

H atoms were positioned geometrically with C—H = 0.93 Å and constrained to ride on their parent atoms with U_iso_(H) = 1.2U_eq_(C).

### Crystal data

**Table:**

C_30_H_20_N_2_	*F*(000) = 856
*M**_r_* = 408.48	*D*_x_ = 1.255 Mg m^−^^3^
Monoclinic, *P*2_1_/*c*	Mo *K*α radiation, λ = 0.71073 Å
Hall symbol: -P 2ybc	Cell parameters from 5640 reflections
*a* = 12.6420 (3) Å	θ = 2.2–22.7°
*b* = 14.8499 (4) Å	µ = 0.07 mm^−^^1^
*c* = 11.8707 (3) Å	*T* = 298 K
β = 104.006 (2)°	Block, pale yellow
*V* = 2162.26 (9) Å^3^	0.2 × 0.2 × 0.2 mm
*Z* = 4	

### Data collection

**Table:**

Bruker SMART 1000 CCD area-detector diffractometer	3128 reflections with *I* > 2σ(*I*)
Radiation source: fine-focus sealed tube	*R*_int_ = 0.038
Graphite monochromator	θ_max_ = 27.5°, θ_min_ = 2.2°
φ and ω scans	*h* = −16→16
35644 measured reflections	*k* = −19→17
4951 independent reflections	*l* = −15→15

### Refinement

**Table:**

Refinement on *F*^2^	Primary atom site location: structure-invariant direct methods
Least-squares matrix: full	Secondary atom site location: difference Fourier map
*R*[*F*^2^ > 2σ(*F*^2^)] = 0.063	Hydrogen site location: inferred from neighbouring sites
*wR*(*F*^2^) = 0.215	H-atom parameters constrained
*S* = 1.33	*w* = 1/[σ^2^(*F*_o_^2^) + (0.1*P*)^2^] where *P* = (*F*_o_^2^ + 2*F*_c_^2^)/3
4951 reflections	(Δ/σ)_max_ < 0.001
289 parameters	Δρ_max_ = 0.20 e Å^−^^3^
0 restraints	Δρ_min_ = −0.20 e Å^−^^3^

### Special details

**Table:**

Geometry. All e.s.d.'s (except the e.s.d. in the dihedral angle between two l.s. planes) are estimated using the full covariance matrix. The cell e.s.d.'s are taken into account individually in the estimation of e.s.d.'s in distances, angles and torsion angles; correlations between e.s.d.'s in cell parameters are only used when they are defined by crystal symmetry. An approximate (isotropic) treatment of cell e.s.d.'s is used for estimating e.s.d.'s involving l.s. planes.
Refinement. Refinement of *F*^2^ against ALL reflections. The weighted *R*-factor *wR* and goodness of fit *S* are based on *F*^2^, conventional *R*-factors *R* are based on *F*, with *F* set to zero for negative *F*^2^. The threshold expression of *F*^2^ > σ(*F*^2^) is used only for calculating *R*-factors(gt) *etc*. and is not relevant to the choice of reflections for refinement. *R*-factors based on *F*^2^ are statistically about twice as large as those based on *F*, and *R*- factors based on ALL data will be even larger.

### Fractional atomic coordinates and isotropic or equivalent isotropic displacement parameters (Å^2^)

**Table:**

	*x*	*y*	*z*	*U*_iso_*/*U*_eq_	
N1	0.56725 (12)	0.37331 (10)	0.11808 (12)	0.0487 (4)	
C18	0.64872 (14)	0.37932 (12)	0.21431 (15)	0.0488 (5)	
C20	0.47454 (15)	0.29870 (13)	−0.05928 (15)	0.0509 (5)	
C10	0.82717 (15)	0.18045 (13)	0.18202 (16)	0.0529 (5)	
N2	0.71173 (15)	0.45087 (13)	0.40278 (15)	0.0651 (5)	
C16	0.65564 (15)	0.24508 (14)	0.05996 (16)	0.0533 (5)	
H16	0.6560	0.2001	0.0056	0.064*	
C17	0.57066 (15)	0.30714 (13)	0.04214 (15)	0.0481 (5)	
C6	0.82357 (16)	0.11106 (13)	0.26209 (17)	0.0551 (5)	
C9	0.90771 (15)	0.18139 (13)	0.11945 (17)	0.0547 (5)	
C15	0.73990 (14)	0.25080 (13)	0.15945 (17)	0.0522 (5)	
C5	0.90315 (17)	0.04023 (14)	0.27789 (19)	0.0619 (6)	
C30	0.47702 (17)	0.24029 (15)	−0.14691 (16)	0.0588 (5)	
H30	0.5407	0.2087	−0.1464	0.071*	
C25	0.63672 (15)	0.44908 (12)	0.29968 (16)	0.0479 (4)	
C8	0.98524 (16)	0.10877 (15)	0.13417 (18)	0.0612 (6)	
C19	0.73694 (15)	0.31995 (13)	0.23649 (17)	0.0547 (5)	
H19	0.7933	0.3269	0.3027	0.066*	
C1	0.74384 (18)	0.10684 (15)	0.32911 (19)	0.0646 (6)	
H1	0.6908	0.1515	0.3199	0.078*	
C29	0.69808 (19)	0.51083 (16)	0.48382 (18)	0.0668 (6)	
H29	0.7490	0.5117	0.5550	0.080*	
C7	0.98102 (17)	0.04122 (15)	0.2135 (2)	0.0675 (6)	
H7	1.0322	−0.0050	0.2239	0.081*	
C13	0.99391 (19)	0.24831 (18)	−0.0222 (2)	0.0722 (6)	
H13	0.9983	0.2948	−0.0733	0.087*	
C14	0.91720 (17)	0.25100 (16)	0.03936 (18)	0.0634 (6)	
H14	0.8692	0.2994	0.0294	0.076*	
C26	0.54987 (18)	0.50785 (16)	0.2783 (2)	0.0708 (6)	
H26	0.4988	0.5066	0.2072	0.085*	
C24	0.38033 (18)	0.34689 (19)	−0.06388 (19)	0.0791 (7)	
H24	0.3781	0.3885	−0.0060	0.095*	
C4	0.8981 (2)	−0.02999 (16)	0.3592 (2)	0.0798 (7)	
H4	0.9490	−0.0764	0.3699	0.096*	
C28	0.6140 (2)	0.56951 (17)	0.4665 (2)	0.0732 (7)	
H28	0.6075	0.6099	0.5243	0.088*	
C2	0.7437 (2)	0.03950 (17)	0.4056 (2)	0.0762 (7)	
H2	0.6917	0.0391	0.4491	0.091*	
C12	1.0679 (2)	0.1750 (2)	−0.0096 (2)	0.0813 (7)	
H12	1.1196	0.1728	−0.0536	0.098*	
C11	1.06313 (19)	0.10878 (18)	0.0663 (2)	0.0780 (7)	
H11	1.1126	0.0615	0.0744	0.094*	
C3	0.8214 (2)	−0.03005 (19)	0.4202 (2)	0.0858 (8)	
H3	0.8197	−0.0764	0.4724	0.103*	
C27	0.5389 (2)	0.56830 (18)	0.3625 (2)	0.0873 (8)	
H27	0.4805	0.6082	0.3485	0.105*	
C22	0.2928 (2)	0.2736 (2)	−0.2388 (2)	0.0911 (9)	
H22	0.2314	0.2637	−0.2989	0.109*	
C21	0.3868 (2)	0.22766 (18)	−0.23564 (19)	0.0789 (7)	
H21	0.3896	0.1872	−0.2947	0.095*	
C23	0.2893 (2)	0.3338 (2)	−0.1538 (2)	0.1001 (10)	
H23	0.2258	0.3662	−0.1561	0.120*	

### Atomic displacement parameters (Å^2^)

**Table:**

	*U*^11^	*U*^22^	*U*^33^	*U*^12^	*U*^13^	*U*^23^
N1	0.0500 (9)	0.0484 (10)	0.0466 (8)	0.0047 (7)	0.0096 (7)	0.0033 (7)
C18	0.0479 (10)	0.0460 (11)	0.0515 (10)	0.0006 (8)	0.0099 (8)	0.0042 (8)
C20	0.0507 (11)	0.0549 (12)	0.0459 (9)	0.0089 (8)	0.0092 (8)	0.0081 (9)
C10	0.0471 (10)	0.0473 (11)	0.0576 (10)	0.0069 (8)	−0.0006 (8)	−0.0051 (9)
N2	0.0652 (11)	0.0657 (12)	0.0604 (10)	0.0019 (9)	0.0072 (8)	−0.0023 (9)
C16	0.0526 (11)	0.0504 (12)	0.0537 (10)	0.0086 (8)	0.0069 (8)	−0.0021 (9)
C17	0.0492 (10)	0.0491 (11)	0.0455 (9)	0.0037 (8)	0.0105 (8)	0.0064 (8)
C6	0.0487 (10)	0.0481 (12)	0.0603 (11)	0.0038 (8)	−0.0028 (8)	−0.0032 (9)
C9	0.0477 (10)	0.0511 (12)	0.0578 (11)	0.0050 (8)	−0.0021 (8)	−0.0067 (9)
C15	0.0483 (10)	0.0449 (11)	0.0603 (11)	0.0059 (8)	0.0069 (8)	0.0028 (9)
C5	0.0532 (12)	0.0502 (12)	0.0728 (13)	0.0069 (9)	−0.0032 (10)	−0.0010 (10)
C30	0.0584 (12)	0.0651 (13)	0.0499 (10)	0.0119 (10)	0.0073 (9)	−0.0045 (10)
C25	0.0503 (10)	0.0412 (10)	0.0519 (10)	−0.0012 (8)	0.0116 (8)	0.0042 (8)
C8	0.0476 (11)	0.0617 (14)	0.0687 (13)	0.0084 (9)	0.0031 (9)	−0.0096 (11)
C19	0.0495 (10)	0.0497 (12)	0.0583 (11)	0.0041 (8)	0.0001 (8)	0.0009 (9)
C1	0.0580 (12)	0.0584 (14)	0.0726 (13)	0.0030 (10)	0.0064 (10)	0.0001 (11)
C29	0.0793 (15)	0.0669 (15)	0.0522 (11)	−0.0039 (11)	0.0117 (10)	−0.0084 (10)
C7	0.0564 (12)	0.0540 (13)	0.0845 (15)	0.0153 (10)	0.0024 (11)	0.0009 (11)
C13	0.0628 (13)	0.0835 (17)	0.0662 (13)	−0.0037 (12)	0.0080 (11)	0.0032 (12)
C14	0.0548 (12)	0.0640 (14)	0.0648 (12)	0.0050 (10)	0.0017 (10)	−0.0022 (11)
C26	0.0701 (14)	0.0677 (15)	0.0673 (13)	0.0178 (11)	0.0026 (10)	−0.0121 (11)
C24	0.0676 (15)	0.1003 (19)	0.0601 (12)	0.0295 (13)	−0.0026 (10)	−0.0151 (13)
C4	0.0747 (16)	0.0581 (15)	0.0971 (17)	0.0133 (12)	0.0022 (14)	0.0174 (13)
C28	0.0850 (16)	0.0680 (15)	0.0690 (14)	0.0044 (12)	0.0233 (12)	−0.0180 (12)
C2	0.0725 (15)	0.0716 (16)	0.0825 (15)	−0.0037 (12)	0.0151 (12)	0.0121 (13)
C12	0.0626 (14)	0.099 (2)	0.0828 (16)	0.0047 (14)	0.0188 (12)	−0.0051 (15)
C11	0.0585 (13)	0.0804 (17)	0.0913 (17)	0.0164 (12)	0.0109 (12)	−0.0061 (15)
C3	0.0856 (18)	0.0694 (17)	0.0977 (18)	−0.0010 (13)	0.0133 (15)	0.0244 (14)
C27	0.0881 (18)	0.0794 (18)	0.0926 (18)	0.0285 (14)	0.0182 (14)	−0.0137 (15)
C22	0.0741 (17)	0.128 (2)	0.0582 (13)	0.0138 (16)	−0.0095 (12)	−0.0087 (15)
C21	0.0836 (17)	0.0906 (18)	0.0563 (12)	0.0096 (14)	0.0047 (11)	−0.0143 (12)
C23	0.0695 (16)	0.150 (3)	0.0691 (15)	0.0432 (17)	−0.0065 (12)	−0.0183 (17)

### Geometric parameters (Å, º)

**Table:**

N1—C17	1.341 (2)	C1—H1	0.9300
N1—C18	1.343 (2)	C29—C28	1.351 (3)
C18—C19	1.396 (3)	C29—H29	0.9300
C18—C25	1.482 (3)	C7—H7	0.9300
C20—C30	1.361 (3)	C13—C14	1.349 (3)
C20—C24	1.379 (3)	C13—C12	1.420 (4)
C20—C17	1.494 (2)	C13—H13	0.9300
C10—C9	1.398 (3)	C14—H14	0.9300
C10—C6	1.410 (3)	C26—C27	1.375 (3)
C10—C15	1.496 (3)	C26—H26	0.9300
N2—C29	1.352 (3)	C24—C23	1.381 (3)
N2—C25	1.355 (2)	C24—H24	0.9300
C16—C15	1.388 (2)	C4—C3	1.344 (4)
C16—C17	1.392 (3)	C4—H4	0.9300
C16—H16	0.9300	C28—C27	1.363 (3)
C6—C1	1.429 (3)	C28—H28	0.9300
C6—C5	1.436 (3)	C2—C3	1.407 (4)
C9—C14	1.429 (3)	C2—H2	0.9300
C9—C8	1.439 (3)	C12—C11	1.345 (4)
C15—C19	1.382 (3)	C12—H12	0.9300
C5—C7	1.385 (3)	C11—H11	0.9300
C5—C4	1.433 (3)	C3—H3	0.9300
C30—C21	1.365 (3)	C27—H27	0.9300
C30—H30	0.9300	C22—C23	1.357 (4)
C25—C26	1.377 (3)	C22—C21	1.363 (4)
C8—C7	1.386 (3)	C22—H22	0.9300
C8—C11	1.415 (3)	C21—H21	0.9300
C19—H19	0.9300	C23—H23	0.9300
C1—C2	1.351 (3)		
			
C17—N1—C18	118.52 (15)	N2—C29—H29	118.4
N1—C18—C19	122.12 (17)	C5—C7—C8	122.16 (19)
N1—C18—C25	116.33 (16)	C5—C7—H7	118.9
C19—C18—C25	121.45 (16)	C8—C7—H7	118.9
C30—C20—C24	118.68 (18)	C14—C13—C12	120.5 (2)
C30—C20—C17	120.31 (17)	C14—C13—H13	119.8
C24—C20—C17	120.95 (18)	C12—C13—H13	119.8
C9—C10—C6	120.79 (17)	C13—C14—C9	121.8 (2)
C9—C10—C15	119.89 (18)	C13—C14—H14	119.1
C6—C10—C15	119.23 (19)	C9—C14—H14	119.1
C29—N2—C25	118.27 (18)	C27—C26—C25	119.7 (2)
C15—C16—C17	119.58 (18)	C27—C26—H26	120.1
C15—C16—H16	120.2	C25—C26—H26	120.1
C17—C16—H16	120.2	C20—C24—C23	120.5 (2)
N1—C17—C16	122.14 (16)	C20—C24—H24	119.7
N1—C17—C20	116.79 (16)	C23—C24—H24	119.7
C16—C17—C20	120.93 (17)	C3—C4—C5	121.3 (2)
C10—C6—C1	123.35 (18)	C3—C4—H4	119.4
C10—C6—C5	119.2 (2)	C5—C4—H4	119.4
C1—C6—C5	117.49 (19)	C29—C28—C27	118.6 (2)
C10—C9—C14	123.36 (18)	C29—C28—H28	120.7
C10—C9—C8	119.35 (19)	C27—C28—H28	120.7
C14—C9—C8	117.3 (2)	C1—C2—C3	120.8 (3)
C19—C15—C16	118.06 (16)	C1—C2—H2	119.6
C19—C15—C10	122.51 (16)	C3—C2—H2	119.6
C16—C15—C10	119.37 (17)	C11—C12—C13	119.7 (2)
C7—C5—C4	122.2 (2)	C11—C12—H12	120.1
C7—C5—C6	119.3 (2)	C13—C12—H12	120.1
C4—C5—C6	118.5 (2)	C12—C11—C8	122.1 (2)
C20—C30—C21	120.5 (2)	C12—C11—H11	118.9
C20—C30—H30	119.7	C8—C11—H11	118.9
C21—C30—H30	119.7	C4—C3—C2	120.4 (2)
N2—C25—C26	120.42 (18)	C4—C3—H3	119.8
N2—C25—C18	117.79 (16)	C2—C3—H3	119.8
C26—C25—C18	121.73 (17)	C28—C27—C26	119.7 (2)
C7—C8—C11	122.3 (2)	C28—C27—H27	120.1
C7—C8—C9	119.2 (2)	C26—C27—H27	120.1
C11—C8—C9	118.6 (2)	C23—C22—C21	119.6 (2)
C15—C19—C18	119.54 (16)	C23—C22—H22	120.2
C15—C19—H19	120.2	C21—C22—H22	120.2
C18—C19—H19	120.2	C22—C21—C30	120.8 (2)
C2—C1—C6	121.5 (2)	C22—C21—H21	119.6
C2—C1—H1	119.3	C30—C21—H21	119.6
C6—C1—H1	119.3	C22—C23—C24	119.8 (2)
C28—C29—N2	123.2 (2)	C22—C23—H23	120.1
C28—C29—H29	118.4	C24—C23—H23	120.1

## References

[bb1] Bruker (2007). *SMART* and *SAINT* Bruker AXS Inc., Madison, Wisconsin, USA.

[bb2] Mi, B.-X., Wang, P.-F., Liu, M.-W., Kwong, H.-L., Wong, N.-B., Lee, C.-S. & Lee, S.-T. (2003). *Chem. Mater.* **15**, 3148–3151.

[bb3] Nishihara, H., Haruna, M. & Suhara, T. (1989). In *Optical Intergrated Circuits* New York: McGraw–Hill.

[bb4] Roberto, D., Ugo, R., Bruni, S., Cariati, E., Cariati, F., Fantucci, P., Invernizzi, I., Quici, S., Ledoux, I. & Zyss, J. (2000). *Organometallics*, **19**, 1775–1788.

[bb5] Sheldrick, G. M. (2008). *Acta Cryst.* A**64**, 112–122. 10.1107/S0108767307043930 18156677

